# P-86. Real Data Comparing Short versus Long Duration of Intravenous Antimicrobial Therapy for Acute Sacral Osteomyelitis

**DOI:** 10.1093/ofid/ofaf695.315

**Published:** 2026-01-11

**Authors:** Hajar AlQahtani, Rakan Sambas, Ahlam AlGhamdi, Nora AlQussair, Leena Alawn

**Affiliations:** National Guard Helath Affairs, Riyadh, Ar Riyad, Saudi Arabia; National Guard Helath Affairs, Riyadh, Ar Riyad, Saudi Arabia; Princess Nourah bint Abdulrahman University, Riyadh, Ar Riyad, Saudi Arabia; National Guard Helath Affairs, Riyadh, Ar Riyad, Saudi Arabia; National Guard Helath Affairs, Riyadh, Ar Riyad, Saudi Arabia

## Abstract

**Background:**

Research on managing patients with sacral osteomyelitis is limited. While longer courses of antimicrobials are common for acute osteomyelitis, a systematic review suggests that a short course (≤ 1 week) may suffice for acute soft tissue infections linked to the ulcer. This study aimed to evaluate the effectiveness of different durations of intravenous antimicrobial therapy for sacral osteomyelitis.

**Methods:**

We conducted a retrospective cohort study on patients diagnosed with sacral osteomyelitis from 2016 to 2021, based on clinical, radiological, and microbiological evidence.

**Results:**

Out of 400 initially screened patients, 80 were confirmed with sacral osteomyelitis. The average age was 70 years, primarily male, with half being malnourished and requiring assistance for daily activities. Fifty percent presented with sepsis, and 20% were bacteremic. Fifty-four percent received intravenous therapy for 30 days, while the rest were treated for 15 days, with 67.5% transitioning to oral antibiotics. Clinical cure rates were similar at 83% for the 30-day group and 90% for the 15-day group. Six patients were admitted to the ICU, with three fatalities. The 30-day and 60-day mortality rates were 4% and 11%, respectively, and 27.5% were readmitted within 60 days for infected sacral wounds. No significant differences in clinical outcomes, complications, or relapse rates were found between the two treatment durations.

**Conclusion:**

Our results suggest that extending intravenous antimicrobial therapy for sacral osteomyelitis does not provide additional benefits.

**Disclosures:**

All Authors: No reported disclosures

